# Rapid discrimination of four *Salmonella enterica* serovars: A performance comparison between benchtop and handheld Raman spectrometers

**DOI:** 10.1111/jcmm.18292

**Published:** 2024-04-23

**Authors:** Quan Yuan, Bin Gu, Wei Liu, Xin‐Ru Wen, Ji‐Liang Wang, Jia‐Wei Tang, Muhammad Usman, Su‐Ling Liu, Yu‐Rong Tang, Liang Wang

**Affiliations:** ^1^ School of Medical Informatics and Engineering Xuzhou Medical University Xuzhou China; ^2^ Department of Laboratory Medicine Shengli Oilfield Central Hospital Dongying China; ^3^ Laboratory Medicine, Guangdong Provincial People's Hospital (Guangdong Academy of Medical Sciences) Southern Medical University Guangzhou China; ^4^ Division of Microbiology and Immunology, School of Biomedical Sciences The University of Western Australia Crawley Western Australia Australia; ^5^ School of Agriculture and Food Sustainability University of Queensland Brisbane Queensland Australia; ^6^ Centre for Precision Health, School of Medical and Health Sciences Edith Cowan University Perth Western Australia Australia

**Keywords:** characteristic peaks, label‐free SERS, machine learning algorithm, Raman spectrometer, Raman spectrum, *Salmonella* serovar

## Abstract

Foodborne illnesses, particularly those caused by *Salmonella enterica* with its extensive array of over 2600 serovars, present a significant public health challenge. Therefore, prompt and precise identification of *S. enterica* serovars is essential for clinical relevance, which facilitates the understanding of *S. enterica* transmission routes and the determination of outbreak sources. Classical serotyping methods via molecular subtyping and genomic markers currently suffer from various limitations, such as labour intensiveness, time consumption, etc. Therefore, there is a pressing need to develop new diagnostic techniques. Surface‐enhanced Raman spectroscopy (SERS) is a non‐invasive diagnostic technique that can generate Raman spectra, based on which rapid and accurate discrimination of bacterial pathogens could be achieved. To generate SERS spectra, a Raman spectrometer is needed to detect and collect signals, which are divided into two types: the expensive benchtop spectrometer and the inexpensive handheld spectrometer. In this study, we compared the performance of two Raman spectrometers to discriminate four closely associated *S. enterica* serovars, that is, *S. enterica* subsp. *enterica* serovar *dublin*, *enteritidis*, *typhi* and *typhimurium*. Six machine learning algorithms were applied to analyse these SERS spectra. The support vector machine (SVM) model showed the highest accuracy for both handheld (99.97%) and benchtop (99.38%) Raman spectrometers. This study demonstrated that handheld Raman spectrometers achieved similar prediction accuracy as benchtop spectrometers when combined with machine learning models, providing an effective solution for rapid, accurate and cost‐effective identification of closely associated *S. enterica* serovars.

## 1 INTRODUCTION

*Salmonella enterica* is a Gram‐negative pathogenetic bacterium of the Enterobacteriaceae family with more than 2600 serotypes.^1^, ^2^ Improper cooking, reheating and handling of food can lead to *Salmonella* outbreaks, and the presence of *Salmonella* in any ready‐to‐eat food should be inhibited. Statistically, food poisoning caused by *Salmonella* ranks first in the world among all types of food pathogens.^3^ However, due to the complex matrix, it is difficult to directly detect the presence of bacterial pathogens in food products. Thus, the continuous development of rapid and accurate methods for detecting *Salmonella* in food has become a key research priority.^4^ Traditional *Salmonella* detection methods consider bacterial culture the gold standard, a time‐consuming and labor‐intensive.^5^ Due to their high accuracy and efficiency, many culture‐independent methods, such as PCR Field (6) and isothermal amplification Field (7), have been suggested recently. However, these methods involve sophisticated procedures of primer designs and experiment operations.^6^ Mass spectrometry shows great potential in rapidly identifying bacterial pathogens in clinical settings.^7^, ^8^ However, it has limitations in discriminating highly similar bacterial species, e.g., *Escherichia coli* and *Shigella* spp., etc.^9^ Therefore, applying the technique in identifying the highly similar and closely associated *S. enterica* serovars is difficult. With the development of sequencing technology, whole‐genome sequencing (WGS) is widely used as a convenient tool for species identification and genotyping.^10^, ^11^ Several 1000s of *Salmonella* genomes have been sequenced and are freely available in public databases, and highly related serovars could be discriminated via bioinformatic methods.^12^ However, the cost of WGS is high, which restricts its routine use in clinical laboratory.^13^ Therefore, developing novel and cheap methods for the rapid and accurate identification of *S. enterica* serovars is crucial.

Raman spectroscopy (RS) is an important all‐biological fingerprinting technique. It has been intensively investigated for its applications in identifying pathogenic microorganisms in recent years due to its generation of information‐rich molecular vibrational spectra.^14^ However, the Raman effect is very weak in practical applications. Later, the development of surface‐enhanced Raman spectroscopy (SERS) greatly improved the magnitude of normal Raman signals.^15^ Therefore, the SERS technique has potential in biomolecular detection due to its high resolution, high sensitivity, low solution interference and strong robustness.^16^ Recent studies have increasingly employed SERS technology to detect microbial pathogens.^17^, ^18^ Yan et al.^19^ applied single‐cell Raman spectrometry for rapid discrimination of 23 bacterial species across 7 genera. The study first utilized decision tree machine learning algorithms to assess and differentiate single bacterial cells at the serotype level and then constructed a quaternary classification model to elevate the accuracy of various recognition models, thus enabling efficient prediction of bacterial strains at the serotype level.^19^ Sun et al.^20^ integrated SERS with convolutional neural network (CNN) models to detect *Salmonella*. By comparing five spectral preprocessing methods, they ultimately identified three Salmonella serotypes at the single‐cell level by combining SG with SNV. Furthermore, Ding et al.^21^ developed a multiscale CNN model with three parallel CNNs by integrating SERS with multiscale convolutional neural networks, achieving multidimensional feature extraction of the SERS spectra, with the model reaching an identification accuracy exceeding 97%. These studies demonstrated that the SERS technique as an analytical tool holds significant potential for rapidly and accurately identifying microorganisms, demonstrating remarkable promise in bacterial detection.

A Raman spectrometer is indispensable to obtain SERS spectra, which are generally categorised into two groups, i.e., expensive benchtop spectrometers and inexpensive handheld spectrometers.^22^ The cost‐effectiveness and user‐friendly nature of portable instruments are attractive, yet their sensitivity and measurement range fall short compared to benchtop setups.^23^, ^24^ However, the ability of portable instruments to facilitate on‐site analysis can overcome the limitations of benchtop instruments. Each type of instrument has its own set of advantages and disadvantages. Therefore, conducting a comparative performance analysis of handheld and benchtop spectrometers is essential to detect bacterial pathogens. Although many studies have applied the SERS technique to rapidly identify bacterial pathogens, fewer studies focus on the performance comparison of handheld and benchtop Raman spectrometers. In this study, we comprehensively compared the performance of a handheld Raman spectrometer (Oceanhood RMS1000 Micro Raman) with a benchtop Raman spectrometer (Renishaw inVia™ Qontor Confocal Raman Microscope) in the detection and prediction of the SERS spectra of four closely‐associated *S. enterica* serovars, namely *S. enterica subsp. enterica serovar dublin*, *enteritidis*, *typhi* and *typhimurium*. According to the results, for SERS spectra generated from both handheld and benchtop Raman spectrometers, different *S. enterica* serovars can be quickly distinguished by various machine learning models, in which the support vector machine (SVM) model consistently achieved the highest accuracies for both spectrometers (99.38% for benchtop Raman spectrometers and 99.97% for handheld Raman spectrometer). In summary, Raman spectroscopy combined with machine learning algorithms can identify closely related *S. enterica* serotypes with high accuracy, which is of great significance for preventing, monitoring and controlling foodborne diseases and protecting food safety. Meanwhile, the results from the comparison of handheld and benchtop Raman spectrometers suggest that the handheld Raman spectrometer can achieve similar prediction accuracy to the benchtop Raman spectrometer in identifying different serotypes of *S. enterica* when combining the technique with machine learning analysis. Therefore, handheld Raman spectrometers could be expected to be more widely used in real‐world scenarios, saving instrument costs for testing laboratories and also helping promote the practical application of in‐field testing via Raman spectroscopy.

## 2 METHODS AND MATERIALS

### 2.1 Bacterial cultivation and sample preparation

Four *S. enterica* serovars were obtained from the Laboratory of the Guangdong Provincial People's Hospital (Guangdong Academy of Medical Sciences), Southern Medical University, China, which includes *S. enterica* subsp. *enterica* serovar *dublin*, *enteritidis*, *typhi* and *typhimurium*. These serovars were preserved in bacterial strain preservation tubes containing a solution of 30% glycerol and 70% TSB broth. All strains were identified and confirmed through biochemical methods plus Matrix‐assisted laser desorption/ionization‐time of flight (MALDI‐TOF) mass spectrometry (MS) and stored at −80°C freezer for long‐term use. During the study, all the strains were recovered from the −80°C freezer by streaking on Columbia blood agar plates (Guangzhou Detgerm Microbiological Science, China) and incubated at 37°C overnight before experimental analysis.

### 2.2 Silver nanoparticle preparation

Preparing silver nanoparticles (AgNPs) has been well‐documented in previous studies.^17^, ^25^, ^26^ In particular, 33.72 mg of silver nitrate (AgNO_3_) was added to a triangular flask containing 200 mL of deionized distilled water (ddH_2_O). The mixture was stirred and heated until it reached the boiling point. Subsequently, 8 mL of sodium citrate (Na_3_C_6_H_5_O_7_) was added while stirring, and the heating was maintained at 650 r/min for 40 min. Afterward, the heating was ceased, and stirring was continued until the solution cooled to room temperature. The solution was then adjusted to a final volume of 200 mL with ddH_2_O. Subsequently, 1 mL of the prepared solution was transferred into a clean Eppendorf (EP) tube and subjected to centrifugation at 7000 r/min for 7 min. Discard the supernatant and resuspend the pellet with 100 μL of ddH_2_O, the AgNP substrate stored without light at room temperature for long‐term use.

### 2.3 SERS spectral collection via benchtop and handheld Raman spectrometers

The benchtop Raman spectrometer used in the experiment is an inVia™ Qontor Confocal Raman Microscope (Renishaw Plc., New Mills, Wotton‐under‐Edge, UK). The microscope had a 785 nm laser, a 1200/mm (514/780) grating, and a charge‐coupled device (Renishaw Centrus 2R4F). The Raman shifts ranged from 500 to 1800 cm^−1^, with an exposure time of 10 s and a laser power of 0.1%. The Raman system was integrated with a microscope (Leica, Germany), and the Raman excitation light was focused onto the sample using a 50× objective lens. The instrument was calibrated using a standard built‐in silicon signal at 520 cm^−1^. In contrast, the handheld Raman spectrometer Oceanhood RMS1000 (Shanghai Oceanhood Opto‐Electronics Tech Co., Ltd., Shanghai, China) was used for sample analysis and Raman spectral detection by using the settings of parameters as excitation wavelength at 785 nm and excitation power at 350 mW. The spectrometer, integrating core components such as lasers, Raman probes, fibre optic spectrometers and photodetectors, forms a compact, high‐performance, research‐grade portable Raman spectrometer. Its highly integrated optical path and structural design afford it a small size and lightweight profile, with a resolution of 12 cm^−1^ at 50 μm. All the SERS spectra were calibrated using the Raman peak at 520 cm^−1^ as the reference peak, and the dark current was deducted during the integration time. For each strain of *Salmonella* used in this study, after recovering via cultivating on a Columbia blood agar plate overnight, a single colony was selected and inoculated into 15 μL phosphate buffer saline (PBS) and well mixed via vortexing, which was then mixed with 15 μL negatively charged AgNPs substrate solution. The well‐mixed suspension was dropped onto the silicon wafer's surface to form a suitable‐sized circular spot, which was dried naturally before SERS spectroscopy. The handheld Raman spectrometer collected a total of 920 spectra, including 4 strains for each serovar of *S. dublin*, *S. enteritidis*, *S. typhi* and *S. typhimurium*, with 230 spectra collected for each *Salmonella* serovar. The benchtop Raman spectrometer collected spectra from 5 strains of *S. dublin*, 9 strains of *S. enteritidis*, 4 strains of *S. typhi* and 10 strains of *S. typhimurium*. Considering the differences in the number of strains of different serovars, 200 spectral data were randomly selected for each serovar for analysis, and a total of 800 spectra were collected for handheld and benchtop Raman spectrometers, respectively.

### 2.4 Average SERS spectra and deconvolution analysis

To compare the performance between the benchtop and handheld Raman spectrometers for *S. enterica* serovars, we computed the average signal intensities of all Raman signals at each Raman shift for a specific sample, generating the average SERS spectra for four closely associated *S. enterica* serovars. This analysis focused on the relative intensity and prevalence of specific peaks in the spectra. Spectral abundance is crucial as it reflects the concentration and presence of various molecular components in the sample. By assessing the abundance of spectral features, we gained insights into each spectrometer's sensitivity and detection capabilities. Additionally, a shaded region representing 20% of the standard deviation (SD) was visualised around the average SERS spectra using Origin Software (OriginLab, United States).^17^ The software's *fit peaks* (pro) function was employed to automatically fit the spectral characteristic peaks, thereby identifying the corresponding molecular components. We performed spectral deconvolution on the average Raman spectra to further explore the differences in spectral data between the two instruments. In the *Voigt* function, convolution using *Lorentzian and Gaussian widths* was used to extract detailed information from each spectral characteristic peak. This approach allowed for a more nuanced analysis of spectral abundance by separating overlapping peaks and elucidating their contributions. The *Gaussian width* and *Lorentzian width* for all characteristic peaks were shared values set to 1, and then convergence was achieved in the fitting process. The comparative analysis of spectral abundance provided additional insights into the performance and detection efficiency of the benchtop and handheld spectrometers for analysing the *S. enterica* serovars.

### 2.5 Clustering analysis of SERS spectra

To investigate the inherent differences of SERS spectra from different instruments and the SERS spectra between *S. enterica* serovars, we employed the Orthogonal Partial Least Squares‐Discriminant Analysis (OPLS‐DA) clustering algorithm.^27^ Since *S. enterica* serovars could be influenced by external factors during the collection process, data normalization was applied as a pre‐processing step in conjunction with cluster analysis to assess data quality. The raw spectral data were normalized using the maximum‐minimum normalization method within the commercial analysis software Unscrambler X (Version 10.4 64bit, CAMO, Norway) to scale all spectral intensities to the range [0, 1]. Cluster analysis was performed using SIMCA's multivariate statistical analysis software (version 13.0, 32‐bit). Select OPLS‐DA as the model type, then click the *Autofit* button to fit the model. The software automatically calculated R2X, R2Y and Q2 to evaluate model performance. SERS spectra from the two Raman spectrometers were first comparatively analysed via clustering analysis. SERS spectral data from different *S. enterica* serovars were then analysed using the same method. SERS spectra from different Raman spectrometers and serovars were represented in distinct colours, and the corresponding data categories were indicated with dashed circles and labels.

### 2.6 Machine learning analysis of SERS spectra

Due to the complexity of SERS spectral data, classical statistical methods are insufficient to analyse the Raman spectral data.^26^ Therefore, machine learning algorithms have been recruited to analyse and predict the identification of the SERS spectrum. To obtain an effective identification model for different *S. enterica* serovars, we compared the performance of six ensemble learning algorithms: Adaptive Boosting (AdaBoost), Decision Tree (DT), Quadratic Discriminant Analysis (QDA), Random Forest (RF), Support Vector Machine (SVM) and eXtreme Gradient Boosting (XGB). Before conducting machine learning (ML) analysis, we employed the *train_test_split* function to split all SERS spectral data into training, validation and test sets in a 6:2:2 ratio. We used the *LabelEncoder* function and the *to_categorical* method in the Scikit‐Learn package (version 0.21.3) to convert the sample labels in the dataset into label‐encoded form. The test dataset was exclusively used to assess the predictive performance of the models and was not utilized for training and validation. During the training of the six machine learning models, we employed grid search functions to train and fine‐tune model parameters. Specifically, the *GridSearchCV* function was used to optimize the hyperparameter combination, and the cv parameter was set to 5, which means that five times of cross‐validation would be performed. The hyperparameter combination with the highest average score was the best for the final model training. We recorded each model's parameter combinations (Table S1) and visualised the gradient model scores (Figure S1).

### 2.7 Evaluation of machine learning models

To validate the ability of machine learning models to distinguish *S. enterica* serovars, quantitative metrics were employed to assess the performance of these algorithms. Common evaluation metrics such as accuracy_score (ACC), precision_score (Pre), recall_score (Recall) and f1_score (F1) have been utilised to gauge the generalisation ability of these models.^28^ The most frequently used evaluation metric is accuracy (ACC), which represents the proportion of correctly classified samples to the total number of samples.^29^ ACC was calculated using the *accuracy_score* function. In order to address potential data imbalances resulting from random data splitting, Precision and Recall were used to provide an objective model evaluation.^30^ The Precision value represents the probability of being correctly predicted as a positive sample among the samples predicted as positive samples in the prediction results. The Recall value represents the probability of being correctly predicted as a positive sample in the positive samples of the original data. During the data analysis process, the average parameters for Precision and Recall were set to Micro and Macro, respectively. As Precision and Recall are mutually influenced indicators, the F1‐Score, as the harmonic average of these two indicators, was calculated for a comprehensive evaluation.^31^ The average parameter for F1‐Score is weighted. To prevent model overfitting during training, we used the 5‐fold cross‐validation (CV) method.^32^ This involved setting cv = 5 in the *cross_val_score* function to split the training dataset into five equal‐sized sub‐datasets. Furthermore, we used the area under the curve (AUC) value, calculated using the *roc_auc_score* function, as a metric to account for class imbalance. To provide a more intuitive visualisation of the model's performance on the test dataset, we employed the *confusion_matrix* function to present the prediction results of the best model in the form of a 4*4 matrix.

### 2.8 Human and animal rights

Not applicable.

## 3 RESULTS

### 3.1 Averaged and deconvoluted SERS spectra

**FIGURE 1:**
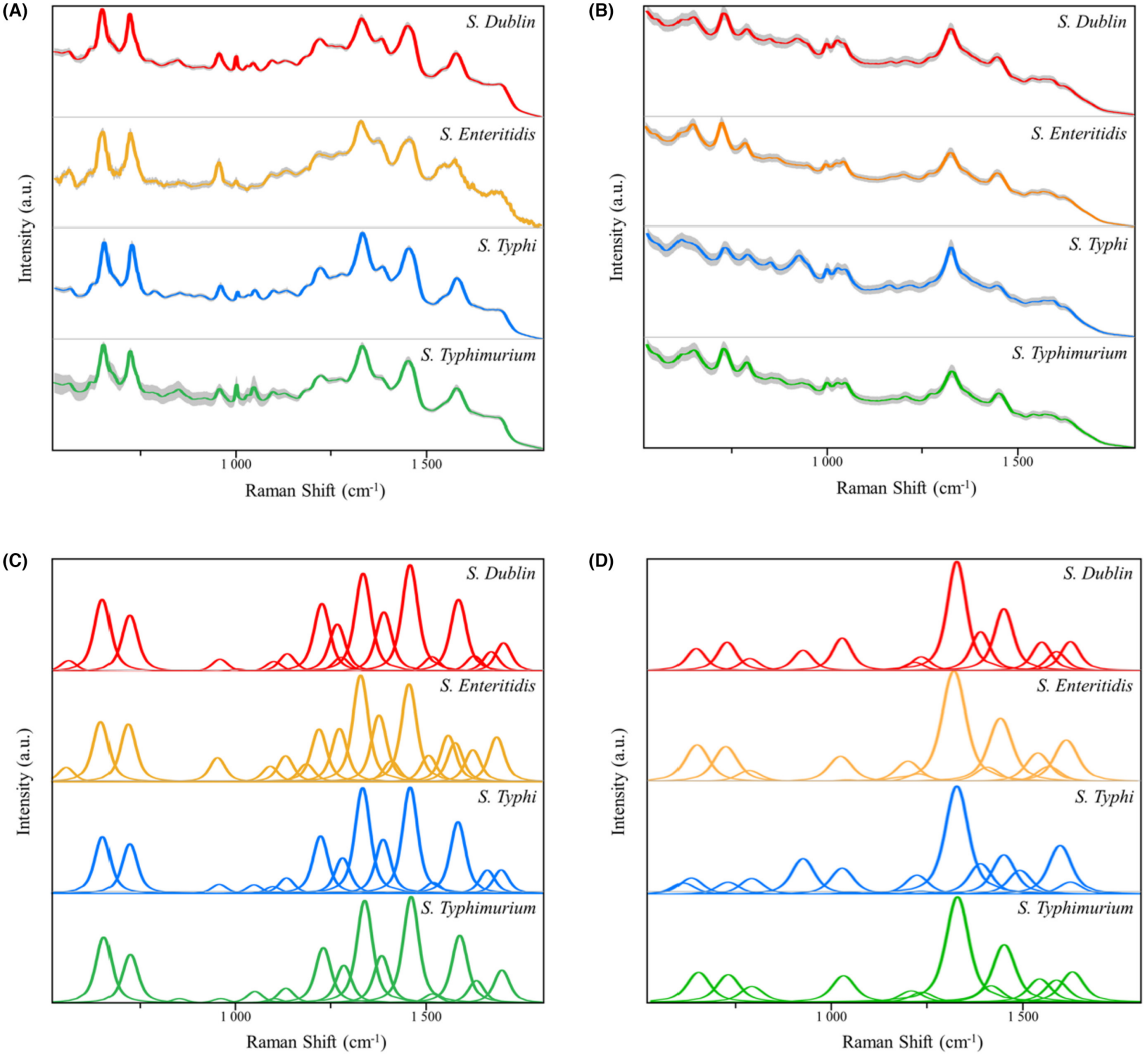
The average and deconvoluted SERS spectra of four *S. enterica* serovars collected from two Raman spectrometers. (A) Average SERS spectra of the four *Salmonella* strains under the benchtop Raman spectrometer. (B) Average SERS spectra of the four *Salmonella* strains under the handheld Raman spectrometer. (C) Deconvoluted SERS spectra of the four *Salmonella* strains under the benchtop Raman spectrometer. (D) Deconvoluted SERS spectra of the four *Salmonella* strains under the handheld Raman spectrometer. The X‐axis represents Raman shifts in the 530–1800 cm^−1^ range, while the Y‐axis represents the relative Raman intensity. a.u. means artificial unit and has no real meaning.

SERS spectra of the same sample collected from different Raman spectrometers vary, which reflects the performance capacity of different spectrometers.^33^ In addition, SERS spectra can reveal variations among different bacterial samples and can transform this chemical and structural information into SERS signal intensities at different Raman shifts.^34^ Therefore, by analysing SERS spectra, the performance of Raman spectrometers can be assessed, and distinct bacterial species can be discriminated. In this study, we collected SERS spectra of four closely related *S. enterica* serovars to compare the differences between benchtop and handheld Raman spectrometers. First, we compared the average SERS spectra of *S. enterica* serovars between the two Raman spectrometers. By comparing the standard errors (SEs) of the average SERS spectra for the four serovars, the overall trend of SERS spectral replicability could be quantified for different SERS spectra.^35^, ^36^ The results indicated that the benchtop Raman spectrometer produced more reproducible SERS spectra than the handheld Raman spectrometer (Figure 1A,B). In general, the reproducibility of SERS spectra generated by both spectrometers varies within an acceptable range. Moreover, comparing the average SERS spectra from the two spectrometers reveals that the benchtop spectrometer's average SERS spectra display more characteristic peaks, likely due to its higher resolution and more sensitive detectors. These factors contribute to the benchtop spectrometer's enhanced ability to detect a wider range of molecular components, resulting in a more abundant spectral representation. However, within each spectrometer, no significant differences were observed in the peak spectra across the four *S. enterica* serovars. Therefore, analysis of the average SERS spectra from variations of standard errors and the number of characteristic peaks could show the performance of different Raman spectrometers but cannot discriminate the four serovars, necessitating more advanced analyses.

To improve the resolution of SERS spectra via enhancement of signal‐to‐noise ratio, spectral deconvolution was conducted. The deconvoluted spectra comprise a series of *Voigt* sub‐bands, each representing a set of characteristic peaks. It could be seen that the method fits the important characteristic peaks of Raman spectra well and could eliminate the interference of other artifactual peaks. The deconvoluted SERS spectra amplify the differences between the four bacterial genera and magnify variations between the two types of spectrometers. Significant disparities exist in the deconvoluted SERS spectra obtained from the two instruments (Figure 1C,D). Specifically, when analysing the deconvoluted SERS spectra for each *Salmonella* strain, the handheld Raman spectrometer displayed fewer characteristic peaks than the benchtop spectrometer. This significant difference can be attributed to the handheld spectrometer's power, sensitivity and resolution limitations. Even with spectral deconvolution analysis, the handheld device cannot discern and display the characteristic peaks, resulting in a substantial disparity with the benchtop spectrometer. Nevertheless, upon closer examination, it becomes apparent that within each spectrometer, there are distinct variations in the deconvoluted SERS spectra for the four *S. enterica* serovars. These differences can be attributed to unique characteristic peaks, allowing for the differentiation and interpretation of the SERS spectra of *S. enterica* serovars.

In specificity, the distinctive SERS spectral characteristic peaks of the four *S. enterica* serovars from different Raman spectrometers and their corresponding chemical structures are presented in Table 1. Characteristic peaks corresponding to the vibrational modes of internal chemical bonds reveal bacterial compositional variations. Due to the complexity of these characteristic peaks, our comparison solely focused on the unique features found between different Raman spectrometers and among the four *S. enterica* serovars. Different *S. enterica* serovars exhibited characteristic peaks at different wavelengths when the same spectrometer was used. Within the same *S. enterica* serovar, the use of different spectrometers also results in the generation of distinct characteristic peaks. In particular, compared to the handheld one, the benchtop spectrometer identifies more molecular bond vibrations for the four *S. enterica* serovars. As indicated in Table 1, measurements obtained with the benchtop spectrometer show that the four *S. enterica* serovars have 20 distinctive characteristic peaks. At the same time, only six were observed when using the handheld spectrometer. Furthermore, only at 1384 cm^−1^, which corresponds to the CN stretching mode and the symmetric CH3 deformation mode,^37^ both *dublin* and *typhi* serovars in the *S. enterica* measured with both the benchtop and handheld Raman spectrometers exhibited identical characteristic peaks. At 1409/1410 cm^−1^, distinctive peaks for *enteritidis* and *typhimurium* can be observed in the handheld spectrometer results, while only the *enteritidis* characteristic peak, corresponding to COO‐ stretching,^38^ is present in the benchtop spectrometer measurements. In addition to these two shared characteristic peaks, for the handheld spectrometer, the four *Salmonella* strains exhibit unique characteristic peaks, including the COO‐ wagging vibrational mode^39^ at 616 cm^−1^, COO‐ and C‐C skeletal stretching^40^ at 925 cm^−1^, Guanine ring mode^41^ at 1486 cm^−1^ and C=C stretching^42^ at 1542 cm^−1^. These characteristic peaks were exclusively present in the measurements obtained with the handheld spectrometer. Conversely, the remaining 18 characteristic peaks from the benchtop spectrometer do not appear in handheld measurements. This suggests significant differences in identifying characteristic peaks between the two spectrometers, with the handheld spectrometer exhibiting noticeably lower sensitivity than the benchtop spectrometer. Additionally, we identified characteristic peaks shared among the *S. enterica* serotypes for the two Raman spectrometers, respectively, which are listed in Table S2. To better understand the differences of the shared characteristic peaks within the four *S. enterica* serovars for handheld and benchtop Raman spectrometers, a set of boxplots was visualised to compare the Raman intensities of these shared characteristic peaks, which are shown in Figure S2.

**TABLE 1:** Four SERS spectral characteristic peaks unique to *Salmonella* measured by benchtop and handheld spectrometers.

No.	Wavenumber (cm^−1^)	Band assignment	Benchtop spectrometer				Handheld spectrometer				*Ref*.
			*S*. D	*S*. E	*S*. T	. Ty	*S*. D	*S*. E	*S*. T	*S*. Ty	
1	568	Deformation mode of the adenosyl ribose ring									43
2	616	COO‐ wagging vibrational mode									39
3	852	C‐O‐C str or ring breathing									44
4	925	COO‐ and C‐C skeletal str									40
5	1004	Phenylalanine									45
6	1049	PO_2_‐ stretching									46
7	1099	CC skeletal and COC stretching									47
8	1128	C‐C str, C‐O‐C									48
9	1134	=C‐C = of unsaturated fatty acids									49
10	1188	Phenyl ring CH, COH bend									50
11	1264	Amide III									51
12	1331	DNA vibration									52
13	1377	Symmetric stretching of the carboxylate									53
14	1384	CN stretching mode and the symmetric CH_3_ deformation mode									37
15	1409/1410	COO‐ stretching									38
16	1486	Guanine ring mode									41
17	1542	C=C stretch									42
18	1557	Amino group in pure chitosan									54
19	1620	Tyrosine									55
20	1651	Amide I									56
21	1664	Amide I									57
22	1683	Amide I									58
23	1690	Amide I									59
24	1696	Amide I									60

### 3.2 Cluster classification of SERS spectra of four *S. enterica* serovars from handheld and benchtop Raman spectrometers

**FIGURE 2:**
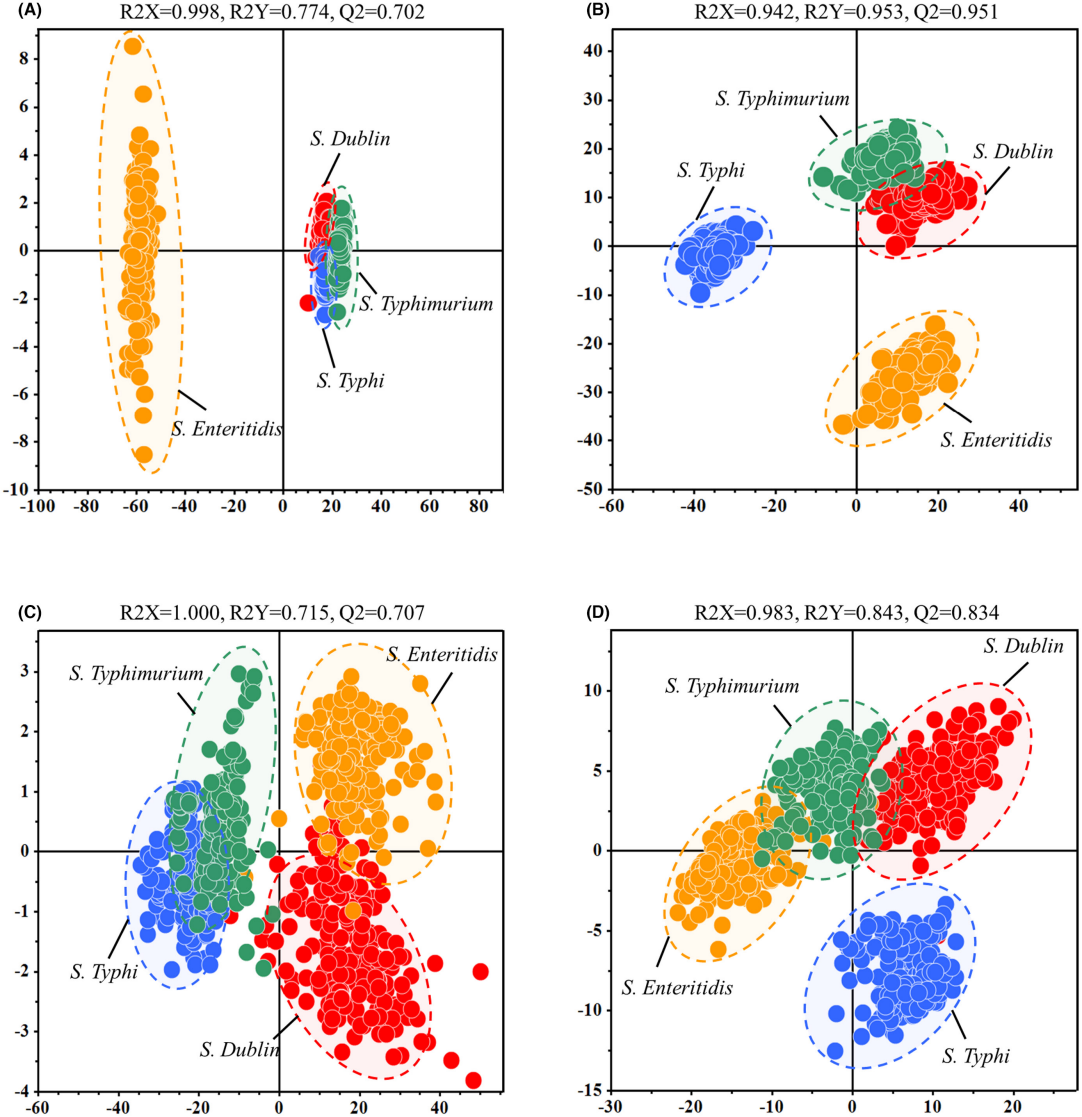
OPLS‐DA analysis of *S. enterica* SERS spectra between the two spectrometers. (A) OPLS‐DA clustering analysis of raw data measured by the benchtop spectrometer. (B) OPLS‐DA clustering analysis of standardized data measured by the benchtop spectrometer. (C) OPLS‐DA clustering analysis of raw data measured by the handheld spectrometer. (D) OPLS‐DA clustering analysis of standardized data measured by the handheld spectrometer.

To identify differences in the generated SERS spectra between different Raman spectrometers and variations in SERS spectra among different *S. enterica* serovars, we utilized the OPLS‐DA clustering algorithm for cluster analysis. We assessed the model's performance using two parameters, R2 and Q2, where R2 reflects the goodness of fit and Q2 reflects the model's predictive capability. A higher Q2 and R2 indicate better clustering effects.^61^ Initially, we imported the raw SERS spectral data measured by the benchtop spectrometer into the OPLS‐DA algorithm (Figure 2A). It can be observed that SERS spectra of *S. enteritidis*, *S. typhimurium* and *S. dublin* obtained with the benchtop spectrometer exhibited a considerable amount of overlap within the same group, with evaluation index scores of R2X = 0.998, R2Y = 0.774 and Q2 = 0.702. After normalising the spectral data, inter‐group differences became more pronounced, leading to improved OPLS‐DA evaluation index scores of R2X = 0.942, R2Y = 0.953 and Q2 = 0.951 (Figure 2B). Figure 2C shows that the raw SERS spectra measured with the handheld spectrometer can effectively differentiate the four *Salmonella* strains, achieving evaluation index scores of R2X = 1.000, R2Y = 0.715 and Q2 = 0.707. Similarly, normalising the spectral data from the handheld spectrometer improves OPLS‐DA evaluation index scores of R2X = 0.983, R2Y = 0.843 and Q2 = 0.834 (Figure 2D). Before the data normalisation, both types of spectrometers exhibited good results in the OPLS‐DA clustering analysis. The benchtop spectrometer showed relatively small differences within the same group compared to the handheld spectrometer's SERS spectra, but inter‐group differences were also limited. After normalizing the spectral data, both spectrometers showed enhanced inter‐group differences, creating clearer group boundaries. In summary, after normalising the spectral data, the benchtop and handheld spectrometers achieved effective clustering analysis for the four *Salmonella* strains. They can differentiate the four strains, but the benchtop spectrometer's SERS spectra exhibit smaller within‐group differences than the handheld spectrometer.

### 3.3 Supervised machine learning analysis of SERS spectra of four *S. enterica* serovars from handheld and benchtop Raman spectrometers

In this study, we constructed and compared six supervised machine learning methods for their ability to differentiate the four *S. enterica* serovars through SERS spectral analysis. In particular, we initially analysed the spectral data based on the benchtop Raman spectrometer. According to the data analysis results in Table 2, the SVM model exhibited the highest predictive accuracy at 99.38%. The Precision, Recall and F1‐Score values for the other three evaluation metrics were 99.38%, 99.44% and 99.38%, respectively. The 5‐fold cross‐validation score was 99.98%, and the AUC value was 99.98%. This suggests that the SVM model outperforms other machine learning algorithms regarding stability and robustness. Apart from the SVM model, the RF, XGBoost, AdaBoost and DT models achieved accuracy rates exceeding 95%, signifying that these models can effectively predict *Salmonella* SERS spectra measured by the benchtop spectrometer with good results.

**TABLE 2:** Comparative analysis of the prediction ability of six machine learning algorithms on *Salmonella* SERS spectral data of benchtop spectrometer.

Algorithm	Accuracy	Precision	Recall	F1‐score	5‐fold CV	AUC
SVM	99.38%	99.38%	99.44%	99.38%	99.98%	99.98%
RF	98.13%	98.13%	98.33%	98.12%	97.97%	98.97%
XGBoost	97.50%	97.50%	97.55%	97.50%	98.28%	98.17%
AdaBoost	97.50%	97.50%	97.55%	97.50%	97.03%	98.00%
DT	96.25%	96.25%	96.44%	96.23%	97.19%	97.12%
QDA	88.75%	88.75%	88.99%	88.97%	86.40%	90.43%

For predicting the four types of *S. enterica* serovars based on SERS spectra acquired with the handheld Raman spectrometer, we also compared six machine learning algorithms, and the results are presented in Table 3. According to the results, the SVM model achieved the highest accuracy, reaching 99.97%. The Precision score was 99.97%, Recall was 99.98%, F1‐Score was 99.97% and AUC was 100%, surpassing the other models. Regarding the 5‐fold cross‐validation score, the SVM model achieved a perfect score of 100%, making it the most robust model among all other machine learning models used in this study. On the other hand, the QDA and AdaBoost algorithms had accuracy rates below 70%, indicating that these two algorithms had relatively poor predictive performance for *S. enterica* serovars based on the analysis of SERS spectra that were acquired with the handheld Raman spectrometer. The observed differences in performance between the models can potentially be attributed to the inherent characteristics of the QDA and AdaBoost algorithms. Specifically, QDA is based on the assumption that the data within each category follows a multivariate normal distribution, and it models the data separately for each class. However, the presence of high noise in Raman spectra can significantly reduce the accuracy of these models. This is because noise can obscure the true spectral features that these algorithms rely on for accurate classification and analysis.^62^ In addition, AdaBoost constructs a strong classifier by sequentially combining multiple weak classifiers. If these weak learners overfit the noise in handheld data, it can lead to lower predictive accuracy.^63^, ^64^ Consequently, handheld spectrometers may generate data with higher variability and noise, causing a decrease in the generalization performance and robustness of QDA and AdaBoost models. In contrast, the SVM, RF and XGB models are more sophisticated, with relatively higher flexibility, stronger adaptability and better tolerance for noise.^65^ Therefore, they maintain a higher level of predictive accuracy when applied to SERS spectral data from the handheld spectrometer.

**TABLE 3:** Comparative analysis of the prediction ability of six machine learning algorithms on the SERS spectral data of *S. enterica* serovars from a handheld spectrometer.

Algorithm	Accuracy	Precision	Recall	F1‐score	5‐fold CV	AUC
SVM	99.97%	99.97%	99.98%	99.97%	99.05%	100%
RF	95.11%	95.11%	95.21%	95.15%	90.90%	95.67%
XGBoost	94.02%	94.02%	94.23%	94.01%	90.22%	95.50%
DT	84.24%	84.24%	84.71%	84.24%	86.97%	85.64%
QDA	64.67%	64.67%	60.21%	63.05%	60.74%	68.62%
AdaBoost	51.63%	51.63%	57.93%	57.05%	61.56%	57.94%

When comparing the performance of machine learning algorithms in predicting different *S. enterica* serovars via SERS spectral analysis from different Raman spectrometers, our research found that the SVM model consistently provided the best predictive results in both instruments, with the handheld spectrometer's predictive results even outperforming those of the benchtop spectrometer. This advantage could be attributed to the robustness of the SVM algorithm to noises and outliers and its effectiveness in handling nonlinear and complex boundaries between categories.^66^, ^67^ Due to the algorithm's enhanced ability to capture nonlinear relationships and latent structures, it can more accurately distinguish between genuine signals and noise. This capability significantly elevates the model's predictive accuracy for handheld Raman spectrometer data, effectively addressing the problem of low data quality associated with handheld Raman spectrometers. However, compared to the other five models, the predictive results of SERS spectra from the handheld spectrometer were noticeably lower than those from the benchtop spectrometer. This suggests that benchtop spectrometers generally offer high‐quality and consistent SERS spectral data compared to handheld spectrometers. The features extracted from benchtop spectrometer data are clearer and more stable, providing an advantage for all algorithms and ensuring that various models consistently maintain high prediction accuracy.

### 3.4 Evaluation of the SVM model

**FIGURE 3:**
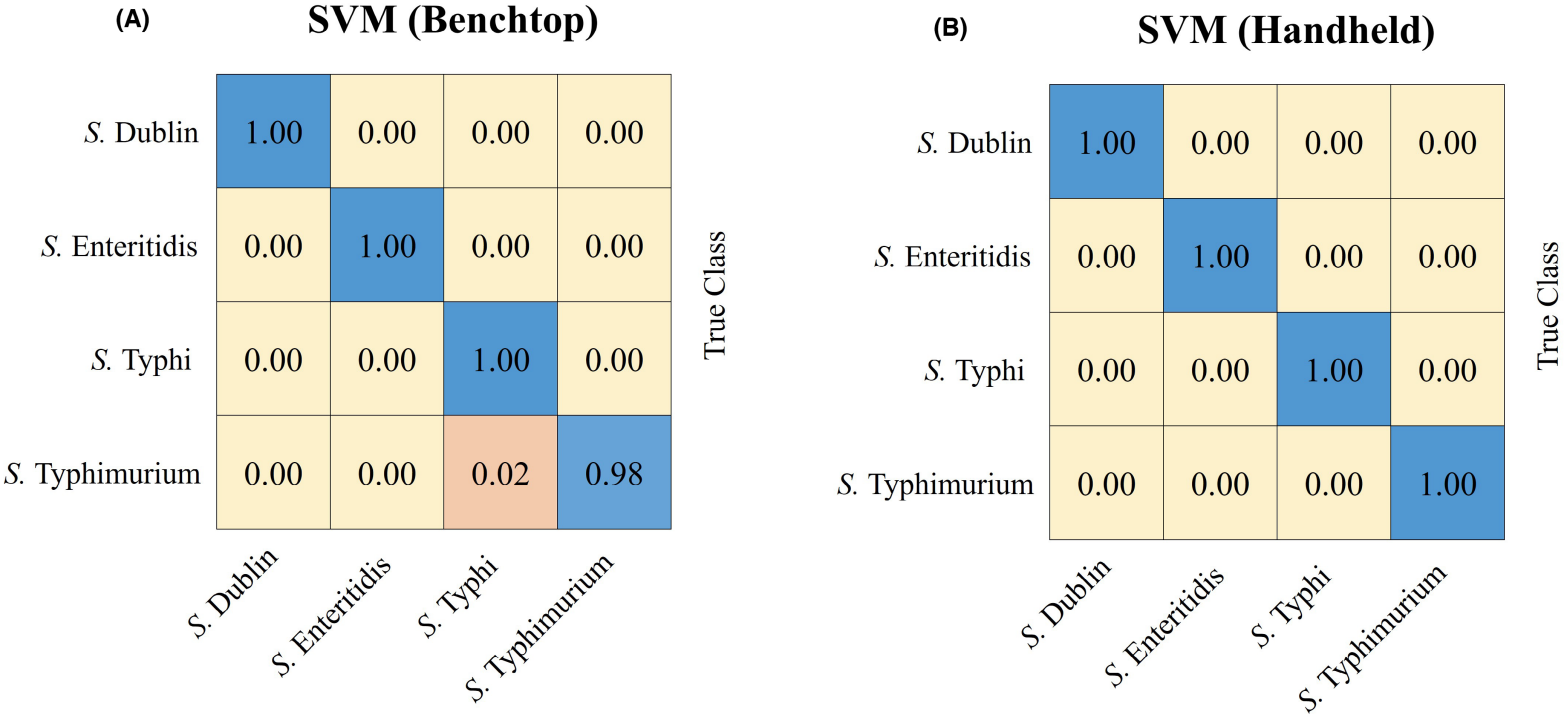
Confusion matrices for the SVM models in the analysis of *S. enterica* SERS spectra generated from benchtop and handheld Raman spectrometers, respectively. (A) Confusion matrix for the SVM model analysis of *S. enterica* SERS spectra from the benchtop Raman spectrometer. An average prediction accuracy of 99.5% was achieved. (B) Confusion matrix for the SVM model analysis of *S. enterica* SERS spectra from the handheld Raman spectrometer. An average prediction accuracy of 100% was achieved. Numbers in the confusion matrix represent the percentage of correctly classified (on the diagonal) or misclassified (off the diagonal) spectra, respectively.

Confusion matrix is typically used to display the classification results of a model, allowing for an intuitive understanding of where the model performs well or poorly on specific samples. In this study, as the SVM model demonstrated the best performance in analysing both the benchtop and handheld spectrometer SERS data, we constructed a confusion matrix for the SVM model. In the confusion matrix, each row represents the model's prediction probabilities for the true samples, and each column represents the model's prediction probabilities for the incorrect samples. Based on the matrix results in Figure 3A, for the benchtop Raman spectrometer's spectral data, the SVM model only incorrectly predicted 2% of *S. typhimurium* samples as *S. typhi*. Moreover, the model achieved 100% prediction accuracy for *S. dublin*, *S. enteritidis* and *S. typhi*. In Figure 3B, the SVM model achieved 100% prediction accuracy for all four Salmonella strains for the handheld Raman spectrometer's spectral data.

## 4 DISCUSSION

Due to the presence of biomacromolecules such as nucleic acids, proteins, lipids and carbohydrates, the SERS spectral differences between bacterial species are subtle, making the distinction of the closely associated serovars within the bacterial species *S. enterica* extremely challenging.^68^ We employed a label‐free SERS technique coupled with machine learning algorithms for differentiation to rapidly and accurately discriminate S. enterica serovars from highly similar SERS spectra. Raman spectroscopy, especially surface‐enhanced Raman spectroscopy, is a promising technique due to its ease of use, cost‐effectiveness, non‐invasiveness and improved signal intensity and quality, holding significant potential for the rapid and precise identification of bacterial pathogens in clinical settings.^69^, ^70^, ^71^ So far, the SERS technique has been extensively explored for its applications in identifying clinically important bacterial pathogens at the genus and species level and in the analysis of bacterial antibiotic resistance.^18^, ^25^, ^72^ There are different types of Raman spectrometers produced by various manufacturers. Generally, the Raman spectrometer is divided into two types: the cheap handheld (portable) version and the expensive benchtop version.^23^ However, few studies currently focus on comparing the performance of benchtop and handheld Raman spectrometers.^22^ Handheld Raman spectrometers have shown promise for on‐site rapid screening due to their portability and ease of use. However, their sensitivity, especially at low pathogen concentrations, tends to be lower compared to benchtop systems.^73^ The detection limits of handheld devices can be affected by multiple factors such as the quality of the SERS substrate, the integration time and the overall design of the spectrometer.^74^ In contrast, benchtop Raman spectrometers, with their more powerful laser sources and sophisticated optical components, generally offer lower detection limits and higher sensitivity, making them more suitable for detecting pathogens at early stages of contamination when the concentration is low.^75^ The application of machine learning algorithms has shown significant potential and advantages in the research field of bacterial species detection and classification. Chen et al. utilised a handheld Raman spectrometer to collect spectral data of four *Salmonella* serovars (*S. enteritidis*, *S. heidelberg*, *S. infantis* and *S. typhimurium*) associated with foodborne outbreaks and used an SVM algorithm to efficiently classify and identify the serovars, with an overall prediction accuracy of over 94%.^76^ Additionally, another study used a benchtop Raman spectrometer to obtain spectral data of three common *S*. serovars, *S. enteritidis*, *S. typhimurium* and *S. paratyphi*. By combining SERS technology with multi‐scale CNN to achieve multi‐dimensional feature extraction from SERS spectral data, the model's recognition accuracy is as high as over 97%.^21^ It is evident that both handheld and benchtop Raman spectrometers are increasingly being integrated with machine learning algorithms, aiming to enhance the precision in the detection and classification of serotype pathogens. In our study, to assess the differences in pathogen detection between the two types of Raman spectrometers, we conducted a comparative analysis of SERS spectra generated from both instruments using the same set of four *S. enterica* serovars.

Because of the inherent variability in Raman spectroscopy, it is necessary to validate the repeatability and uniformity of the SERS spectra. In this study, to verify the reproducibility of SERS spectra, we calculated the average signal intensities and standard errors along with their corresponding Raman shifts. The comparison of average SERS spectra revealed notable differences between the two Raman spectrometers for the same set of the four *S. enterica* serovars. In particular, the benchtop Raman spectrometer, benefiting from their higher resolution and sensitivity, produced SERS spectra with flatter baselines and more characteristic peaks. This can be attributed to their superior optical and electronic components, which enhance measurement precision and reduce signal noise and distortion. Additionally, the greater stability and consistency in measurements afforded by the benchtop spectrometer's robust design and controlled operating environment contributed to reduced variability in the spectra, as evidenced by smaller standard error bands. Conversely, the handheld Raman spectrometer, designed specifically for portability, displayed SERS spectra with greater variability. These instruments' compact size and lightweight nature may increase sensitivity to environmental factors, leading to fluctuations in baselines and standard error bands. Additionally, the handheld spectrometer has sensitivity and calibration accuracy limitations, affecting the overall quality and uniformity of the collected SERS spectra.

Regarding the SERS spectral clustering analysis, the OPLS‐DA algorithm, a supervised discriminant analysis technique, effectively mitigates the impact of inter‐group differences on classification outcomes.^70^, ^77^ It achieves the effects by reducing model overfitting and the probability of false positives through an orthogonal signal processing.^78^ In this study, we applied the OPLS‐DA method to classify the SERS spectra of four *S. enterica* serovars. The results indicated that, before data normalisation, the OPLS‐DA clustering outcomes from both spectrometers could distinguish among different *S. enterica* serovars. Following SERS data normalization, the inter‐group differences increased while intra‐group differences decreased. This normalisation led to improved R2X, R2Y and Q2 scores for the SERS spectra from both Raman spectrometers. However, we found that the scores obtained from the benchtop spectrometer data improved more significantly than those from the handheld spectrometer. This enhancement can be attributed to the inherently higher resolution and sensitivity of benchtop spectrometers, which enable more subtle spectral features to be detected. Consequently, during the normalisation process, these minute features are better preserved and emphasised, enhancing the model's variance explanation capability and predictive performance.^79^, ^80^ Furthermore, handheld spectrometers may be more susceptible to higher levels of noise interference. In contrast, benchtop spectrometers produce less noise with their precise optical components and stable operating environments. This results in the normalisation of data that more accurately reflects true spectral characteristics, leading to a greater improvement in the scores of the benchtop spectrometer data by the OPLS‐DA algorithm.^81^

In this study, we also compared the prediction capacity of machine learning modes on the SERS spectra from different Raman spectrometers. Machine learning, recognised as a powerful data analysis method, excels in exploring local features and extracting global characteristics from signal data.^30^, ^82^ Leveraging its immense potential in SERS spectral data processing and analysis, it has found its applications in rapidly identifying and predicting bacterial genera and species.^19^, ^83^ We constructed six supervised machine learning models to identify the algorithm with optimal predictive performance (AdaBoost, DT, QDA, RF, SVM and XGB). We applied them to SERS spectral data from benchtops and handheld Raman spectrometers. The evaluation was performed through various predictive parameters and confusion matrices. The results demonstrated that SVM exhibited the best computational accuracy and robust performance in handling complex classification problems. It achieved the highest accuracy in handheld and benchtop Raman spectrometers, indicating that integrating SERS technology with machine learning algorithms facilitates the rapid and accurate identification of *S. enterica* serovars, independent of the Raman spectrometer types. This underscores its immense potential in bacterial pathogen detection and food safety monitoring. Furthermore, the handheld Raman spectrometer has demonstrated sufficient accuracy and robustness in identifying *S. enterica* serovars that is comparable to the expensive benchtop Raman spectrometer, suggesting their potential as an effective alternative in scenarios requiring instrument portability, mobility and cost‐efficiency, thereby presenting possibilities for widespread application in the field of public health safety.

In addition, in real‐world settings, it is often necessary to process mixed samples containing multiple bacterial serotypes, which will face challenges such as spectral overlap, background signal interference and low signal intensity differences. Especially when trying to differentiate between pathogens with very similar cell wall composition and metabolic characteristics, SERS substrate‐based detection and identification methods are more challenging.^4^, ^84^ Chen et al.^76^ used a portable Raman spectrometer to detect six bacterial mixed samples containing four *Salmonella* serovars, *Staphylococcus aureus* and *Escherichia coli* and found that the overall accuracies predicted by both linear discriminant analysis (LDA) and SVM models were lower than those assessed by the stand‐alone pure cultures, with the lowest accuracy being only 65%. One of the reasons for this decrease in accuracy is due to the highly homogeneous structure of the cell surface, which limits the classification ability of serotyping. However, the differences found between pure cultures and bacterial mixtures suggest that alterations in bacterial metabolism during mixed cultures may be a more important reason.^85^ Martinez et al.^84^ utilised a benchtop Raman pectrometer in combination with SERS spectroscopy and multivariate analysis methods for label‐free detection and identification of single bacteria in a mixture of bacteria. The SERS platform demonstrated its ability to differentiate between Gram‐positive and Gram‐negative bacteria and to identify samples containing different concentrations of these two groups of bacteria. By applying multivariate spectral analysis techniques such as PCA and PLS‐DA, the spectra of pure samples showed significant clustering properties. As the concentration of one bacterium in the mixed sample increased and the concentration of the other decreased, the mixed population shifted towards the pure sample with the dominant bacterium, resulting in a decrease in the model's classification ability.^84^ In summary, both handheld and benchtop spectrometers face substantial challenges in identifying mixed bacterial samples. The primary reason lies in the highly homologous structures on the surfaces of pathogens with similar cell wall compositions and metabolic profiles.^86^, ^87^ In practical applications such as food safety monitoring, disease diagnosis and environmental assessment, the detection of mixed bacterial samples presents a common and complex challenge.^87^ Therefore, it is necessary to develop integrated methods with advanced computational models and optimise standard operating procedures to enhance the sensitivity, accuracy and reproducibility of SERS technique in detecting mixed bacteria.

## 5 CONCLUSION

In this study, we conducted a performance comparison of handheld and benchtop Raman spectrometers in detecting *S. enterica* serovars via machine learning analysis of SERS spectra. The benchtop Raman spectrometer, leveraging its high sensitivity and resolution, provided more comprehensive Raman spectral information in laboratory analysis. Conversely, while lacking capacity in characteristic peak detection, the handheld Raman spectrometer demonstrated significant advantages in portability, mobility and cost‐effectiveness, making it invaluable for rapid on‐site screening. Regardless of whether a benchtop or handheld device was used, both instruments achieved high accuracy in differentiating four *S. enterica* serovars through supervised clustering analysis. By optimizing data processing parameters of a set of machine learning algorithms, especially in conjunction with SVM models, we confirmed that the handheld Raman spectrometer could achieve sufficient accuracy in *S. enterica* serovar detection even in resource‐limited environments, with its predictive accuracy slightly surpassing that of the benchtop Raman spectrometer. This outcome further underscores the potential of Raman spectroscopy technology, coupled with machine learning algorithms, for rapidly and accurately identifying closely related bacterial pathogens. Future research directions should include further optimising the performance of handheld Raman spectrometers, enhancing the quality of spectral signal acquisition, refining data processing algorithms and exploring SERS substrates more suitable for on‐site detection. These efforts aim to ensure handheld Raman spectrometers' practicality, reliability and applicability in food safety control and monitoring. In conclusion, this study demonstrates that the combination of SERS spectroscopy and machine learning algorithms enables the rapid identification of very similar *S. enterica* serovars, offering a new technological approach for the swift detection of *Salmonella*. In addition, the handheld Raman spectrometer exhibits similar accuracy to a benchtop Raman spectrometer in identifying *S. enterica* serovars. This finding significantly enhances its practical value in on‐site rapid detection. As the handheld Raman spectroscopy technology progresses and optimises, it promises to achieve economically efficient applications across diverse fields.

**Table:** 

Software list				
Software	Version	Company	Country	Link
Origin	2021	OriginLab	United States	https://www.originlab.com/
Unscrambler X	10.4	CAMO	Norway	https://www.pharmaceutical‐technology.com/products/unscrambler‐x/
SIMCA	13.0	Umetrics	Sweden	https://www.sartorius.com/en
PyCharm	2023.1.3	JetBrains	Czech Republic	https://www.jetbrains.com/pycharm/
Scikit‐Learn	0.21.3	Open Source Community	–	https://scikit‐learn.org/stable/

## AUTHOR CONTRIBUTIONS

**Quan Yuan:** Visualization (equal); writing – original draft (equal). **Bin Gu:** Writing – original draft (equal). **Wei Liu:** Writing – original draft (equal). **Xin‐Ru Wen:** Writing – original draft (equal). **Ji‐Liang Wang:** Writing – original draft (equal). **Jia‐Wei Tang:** Writing – original draft (equal). **Muhammad Usman:** Writing – original draft (equal). **Su‐Ling Liu:** Writing – original draft (equal). **Yu‐Rong Tang:** Writing – original draft (equal). **Liang Wang:** Funding acquisition (equal); writing – original draft (equal); writing – review and editing (equal).

## CONFLICT OF INTEREST STATEMENT

The authors declare that the research was conducted in the absence of any commercial or financial relationships that could be construed as a potential conflict of interest.

## INCLUSION AND DIVERSITY

We support inclusive, diverse and equitable conduct of research.

## Supporting information


Data S1.

## Data Availability

The raw data supporting the conclusions of this article will be available under request without undue reservation.
