# Supplementary material for: Rapid discrimination of four Salmonella enterica serovars: A performance comparison between benchtop and handheld Raman spectrometers
Source: J Cell Mol Med. 2024 Apr 23;28(8):e18292. doi: 10.1111/jcmm.18292 (PMC11037414; doi:10.1111/jcmm.18292)
Supplement: Supplementary file 1 — Data S1. [file JCMM-28-e18292-s001.docx]

**Supplementary Files**

**
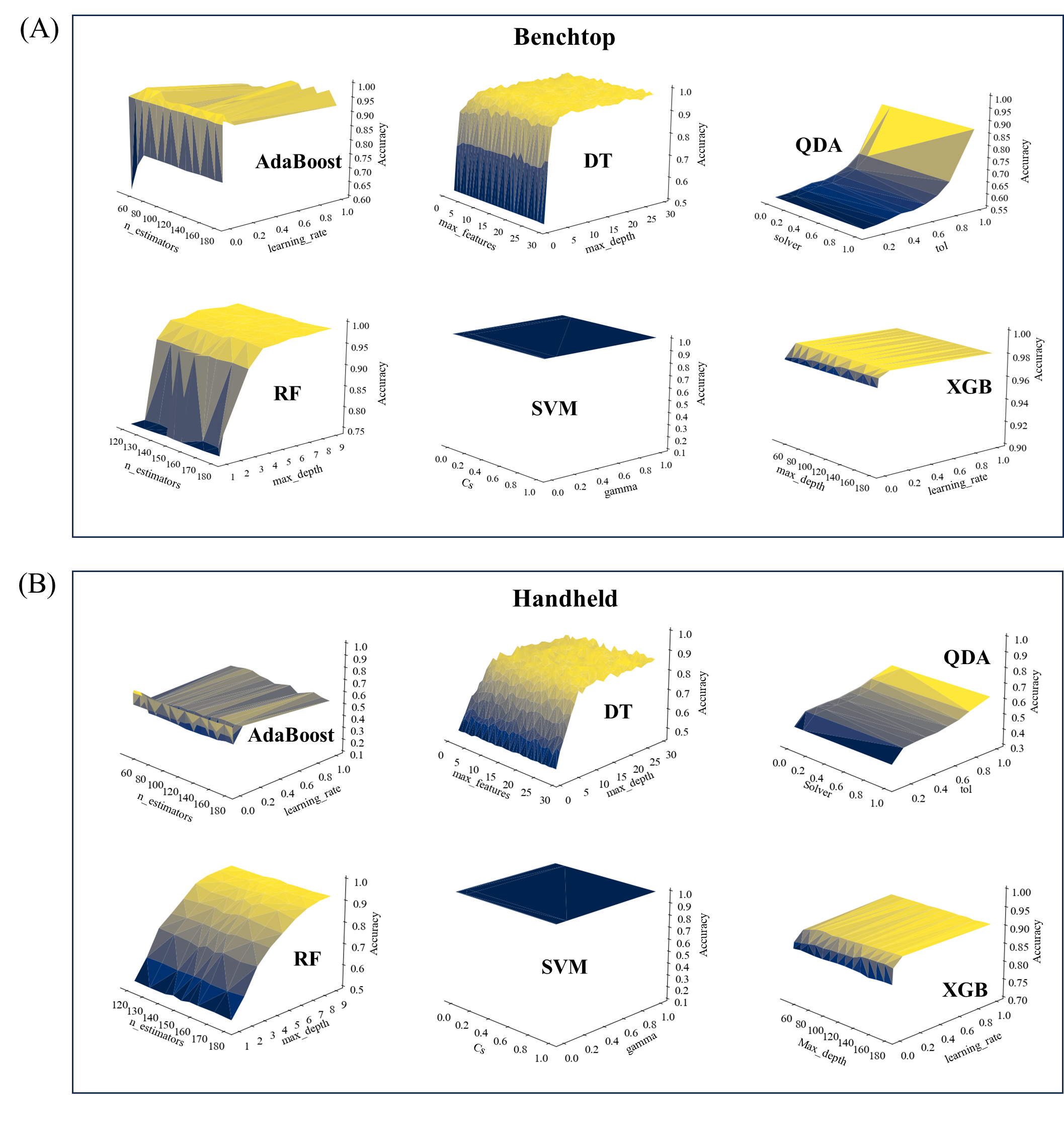
**

**Supplementary Figure S1** Parameter optimization of six supervised machine learning algorithms used in this study illustrates the discrimination accuracy rates of ensemble learning models throughout the parameter combination and iteration process. (A) Six machine learning models for benchtop Raman spectrometer: AdaBoost, DT, QDA, RF, SVM, and XGB. (B) Six machine learning models for handheld Raman spectrometer: AdaBoost, DT, QDA, RF, SVM, and XGB. The vertical axes in all figures represent the discrimination accuracy rates of ensemble learning models during the parameter combination and iteration process.

**
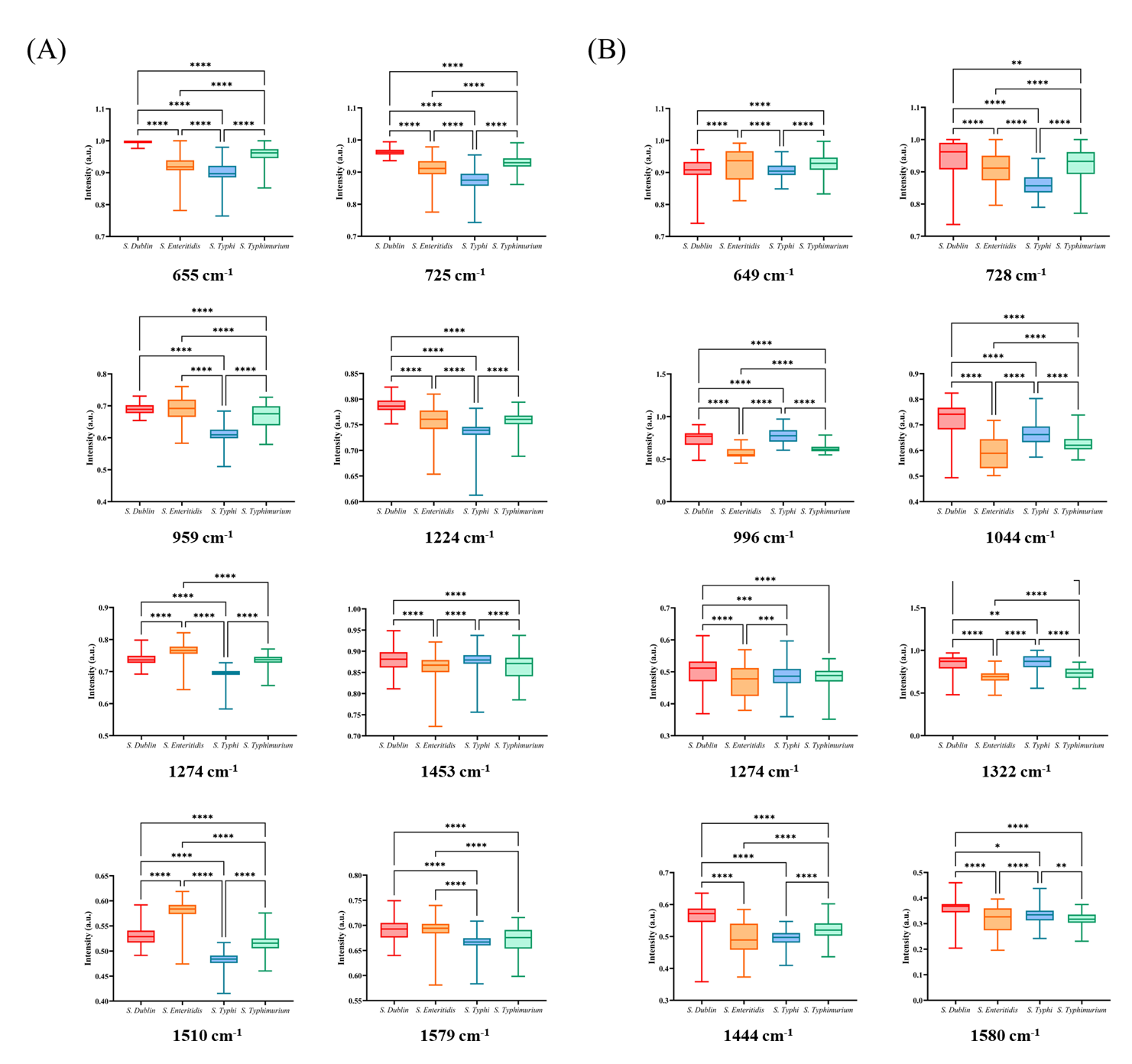
**

**Supplementary Figure S2** Boxplots of the shared characteristic peaks of the four Salmonella strains under measurements from both spectrometers. (A) Boxplots comparing 8 selected shared characteristic peaks under benchtop spectrometer measurements. (B) Boxplots comparing 8 selected shared characteristic peaks under handheld spectrometer measurements. The difference analysis method used for each set of data is one-way ANOVA.

**Supplementary Tables**

**Supplementary Table S1** The best combination of hyperparameters for different machine learning algorithms used to construct prediction models via SERS spectra Benchtop and Handheld Spectrometer

| **Algorithms** | **Function** | **Parameter Range** | **Optimum Parameter** | |
| --- | --- | --- | --- | --- |
|  |  |  | **Benchtop Spectrometer** | **Handheld**  **Spectrometer** |
| **AdaBoost** | AdaBoost  Classifier | **learning_rate** = [0.1, 1, 0.01, 0.001],  **n_estimators** = [50, 60, 70, 80, 90, 100, 110, 120, 130, 140, 150, 160, 170, 180] | **learning_rate** = 0.01, **n_estimators** = 180 | **learning_rate** = 0.01, **n_estimators** =  60 |
| **DT** | DecisionTree  Classifier | **criterion** = ['gini', 'entropy'],  **max_depth** = range (1, 30), **max_features** = [21, 22, 23, 24, 25, 26, 28, 29, 30, 'auto'] | **criterion** = 'entropy',  **max_depth** = 22,  **max_features** = 23 | **criterion** = 'gini',  **max_depth** = 28,  **max_features** = 26 |
| **QDA** | Quadratic  Discriminant  Analysis | **reg_param =** np.linspace(0, 1, 10),  **tol =** [0.0001, 0.001, 0.01, 0.1, 1] | **reg_param =** 1.0,  **tol =** 0.0001 | **reg_param =** 0.8,  **tol =** 0.0001 |
| **RF** | RandomForestClassifier | **Criterion** = ['gini', 'entropy'],  **max_depth** = range (1, 10),  **n_estimators** = [50, 60, 70, 80, 90, 100, 110, 120, 130, 140, 150, 160, 170, 180] | **criterion** = 'entropy', **max_depth** = 7, **n_estimators** = 155 | **criterion** =  'gini',  **max_depth** = 9, **n_estimators** = 150 |
| **SVM** | SVC | **Cs =** [0.0001, 0.001, 0.01, 0.1, 1, 2, 3, 4, 5, 10],  **gamma =** [0.0001, 0.001, 0.01, 0.1, 1],  kernel = ['rbf', ' linear '] | **Cs =** 0.001,  **gamma =** 0.001,  **kernel =** 'linear' | **Cs =** 0.001,  **gamma =** 0.001,  **kernel =** 'linear' |
| **XGB** | XGB  Classifier | **n_estimators =** [50, 60, 70, 80, 90, 100, 110, 120, 130, 140, 150, 160, 170, 180],  **learning_ rate =** [0.1, 1, 0.01, 0.001] | **Estimators** = 50, **learning_rate** = 0.1 | **Estimators** = 50, **learning_rate** = 1 |

**Supplementary Table S2** Four SERS spectral characteristic peaks shared to *Salmonella* measured by benchtop and handheld spectrometers.

| **Wavenumber (cm^-1^)** | **Band Assignment** | **Benchtop Spectrometer** | | | | **Handheld Spectrometer** | | | | ***Ref.*** |
| --- | --- | --- | --- | --- | --- | --- | --- | --- | --- | --- |
|  |  | ***S.* D** | ***S.* E** | ***S.* T** | ***S.* Ty** | ***S.* D** | ***S.* E** | ***S.* T** | ***S.* Ty** |  |
| 649 | Amide I |  |  |  |  |  |  |  |  | 1 |
| 655 | Guanine |  |  |  |  |  |  |  |  | 2 |
| 725 | Adenine or adenine-containing molecules |  |  |  |  |  |  |  |  | 3 |
| 728 | Adenine |  |  |  |  |  |  |  |  | 4 |
| 787 | Cytosine or uracil |  |  |  |  |  |  |  |  | 5 |
| 959 | C=C deformation or C–N stretching |  |  |  |  |  |  |  |  | 6 |
| 996 | C-C stretching |  |  |  |  |  |  |  |  | 7 |
| 1026 | C=C stretching vibration |  |  |  |  |  |  |  |  | 8 |
| 1044 | C-O/C-N stretching vibrations |  |  |  |  |  |  |  |  | 9 |
| 1213 | C-H in-plane bending of phenylalanine andor tyrosine |  |  |  |  |  |  |  |  | 10 |
| 1224 | Amide III |  |  |  |  |  |  |  |  | 11 |
| 1230 | Amide III |  |  |  |  |  |  |  |  | 12 |
| 1274 | Amide III |  |  |  |  |  |  |  |  | 13 |
| 1322 | Amide III |  |  |  |  |  |  |  |  | 14 |
| 1444 | δCH2 lipids and fatty acids |  |  |  |  |  |  |  |  | 15 |
| 1453 | -CH 2 bending |  |  |  |  |  |  |  |  | 16 |
| 1510 | C = C str carotenoids |  |  |  |  |  |  |  |  | 17 |
| 1579/1580 | Adenine, thymine, guanine |  |  |  |  |  |  |  |  | 18 |
| 1616 | Tyrosine, (C-N) stretching vibration |  |  |  |  |  |  |  |  | 5 |

**References**

1. Shi-jie L, Yao-di Z, Miao-yun L, et al. Raman Spectroscopic Characteristic Structure Analysis and Rapid Identification of Food-Borne Pathogen Spores Based on SERS Technology. 2022;42(9):2774-2780.

2. Dina NE, Colniță A, Leopold N, Haisch CJPt. Rapid single-cell detection and identification of bacteria by using surface-enhanced raman spectroscopy. 2017;27:203-207.

3. Yang D, Zhou H, Dina NE, Haisch C. Portable bacteria-capturing chip for direct surface-enhanced Raman scattering identification of urinary tract infection pathogens. *R Soc Open Sci*. Sep 2018;5(9):180955. <https://doi.org10.1098/rsos.180955>.

4. Jin H, Wang J, Jin S, Jiang L, Zou YJSAPAM, Spectroscopy B. Raman spectroscopy of potential bio-hazards commonly found in bio-aerosols. 2020;243:118753.

5. Zhu Y, Liu S, Li M, et al. Preparation of an AgNPs@ Polydimethylsiloxane (PDMS) multi-hole filter membrane chip for the rapid identification of food-borne pathogens by surface-enhanced Raman spectroscopy. 2022;267:120456.

6. Bashir S, Nawaz H, Majeed MI, et al. Rapid and sensitive discrimination among carbapenem resistant and susceptible E. coli strains using Surface Enhanced Raman Spectroscopy combined with chemometric tools. *Photodiagnosis Photodyn Ther*. Jun 2021;34:102280. <https://doi.org10.1016/j.pdpdt.2021.102280>.

7. Aneeba B, Ashvin Santhia SV, Vinu S, Christy RS, Al Farraj DA, Alkubaisi NA. Influence of most reactive inorganic cation in the optical and biological activities of L-Lysine monohydrochloride crystal. *Saudi J Biol Sci*. Nov 2020;27(11):2961-2967. <https://doi.org10.1016/j.sjbs.2020.07.018>.

8. Yao X, Lin J, Zhou Q, et al. A new platform for rapid and indiscriminate detection of environmental pollutants based on surface-enhanced Raman spectroscopy. *Environmental Science: Nano*. 2023;10(9):2374-2386. <https://doi.org10.1039/d3en00461a>.

9. Nakar A, Wagenhaus A, Rösch P, Popp JJA. Raman spectroscopy for the differentiation of Enterobacteriaceae: a comparison of two methods. 2022;147(17):3938-3946.

10. Kamińska A, Witkowska E, Kowalska A, et al. Highly efficient SERS-based detection of cerebrospinal fluid neopterin as a diagnostic marker of bacterial infection. 2016;408:4319-4327.

11. Saraeva I, Zayarny D, Tolordava E, et al. Locally Enhanced Electric Field Treatment of E. coli: TEM, FT-IR and Raman Spectrometry Study. 2023;11(7):361.

12. Lippert J, Tyminski D, Desmeules PJJotACS. Determination of the secondary structure of proteins by laser Raman spectroscopy. 1976;98(22):7075-7080.

13. Hsu B-L, Weng Y-M, Liao Y-H, Chen WJJoA, Chemistry F. Structural investigation of edible zein films/coatings and directly determining their thickness by FT-Raman spectroscopy. 2005;53(13):5089-5095.

14. Tay L-L, Tanha J, Ryan S, Veres T. Detection of Staphylococci aureus cells with single domain antibody functionalized Raman nanoparobes. Photonics North 2007: SPIE; 2007. p. 101-107.

15. Lu X, Rasco BA, Jabal JM, et al. Investigating antibacterial effects of garlic (Allium sativum) concentrate and garlic-derived organosulfur compounds on Campylobacter jejuni by using Fourier transform infrared spectroscopy, Raman spectroscopy, and electron microscopy. 2011;77(15):5257-5269.

16. Saleem M, Majeed MI, Nawaz H, et al. Surface-enhanced Raman spectroscopy for the characterization of the antibacterial properties of imidazole derivatives against bacillus subtilis with principal component analysis and partial least squares–discriminant analysis. 2022;55(13):2132-2146.

17. Rösch P, Harz M, Schmitt M, et al. Chemotaxonomic identification of single bacteria by micro-Raman spectroscopy: application to clean-room-relevant biological contaminations. 2005;71(3):1626-1637.

18. Lorenz B, Rösch P, Popp JJA, chemistry b. Isolation matters—processing blood for Raman microspectroscopic identification of bacteria. 2019;411:5445-5454.
